# Acupuncture treatment for anti-NMDAR encephalitis: A case report

**DOI:** 10.1097/MD.0000000000038546

**Published:** 2024-06-21

**Authors:** Tianhong Yang, Hui Cheng, Chunlei Gu

**Affiliations:** a Department of Acupuncture and Moxibustion, The First Affiliated Hospital of Zhejiang Chinese Medical University, Hangzhou, China; b Department of Pediatric Critical Care Rehabilitation Unit, The First Affiliated Hospital of Zhejiang Chinese Medical University, Hangzhou, China.

**Keywords:** acupuncture, case report, encephalitis

## Abstract

**Rationale:**

Anti-N-methyl-D-aspartate receptor (anti-NMDAR) encephalitis is a rare disease and common type of autoimmune encephalitis. The prognosis of patients with comorbid disorders of consciousness is poor, and no such acupuncture treatment has been reported. We report a case of acupuncture in anti-NMDAR encephalitis with a high cerebrospinal fluid titer combined with impaired consciousness.

**Patient concerns:**

A 13-year-old girl with anti-NMDAR encephalitis presented to our hospital with impaired consciousness.

**Diagnoses:**

Therefore, the patient was diagnosed with anti-NMDAR encephalitis. According to the Chinese medicine theory, the diagnosis was Shenhun(phlegm obstructs the clear orifices).

**Interventions:**

Depending on the patient’s condition, we used the Xingnao Kaiqiao acupuncture therapeutic method.

**Outcomes:**

After 16 weeks of acupuncture treatment, the patient awoke and resumed a normal life with no recurrence at one-year follow-up.

**Conclusion:**

This case demonstrated that acupuncture can be used as a complementary and alternative treatment for anti-NMDAR encephalitis.

## 1. Introduction

Anti-N-methyl-D-aspartate receptor (anti-NMDAR) encephalitis is an autoimmune encephalitis first proposed by Dalmau in 2007.^[1]^ Patients mainly present with impaired consciousness, mental abnormalities, memory disorders, autonomic dysfunction, involuntary movements, etc. The etiology of anti-NMDAR encephalitis remains unclear, and it is believed to be related to viral infections and tumors.^[2]^ In treatment, the main focus is on immunomodulation or immunosuppression of immune function, and symptomatic therapy is administered simultaneously. Acupuncture treatment is a traditional therapy in Chinese medicine and plays an important role in the promotion of wakefulness owing to its safety, convenience, and effectiveness. There are many reports on anti-NMDAR encephalitis in China, but there are no reports on acupuncture treatment in patients with high titers combined with impaired consciousness.

## 2. Case presentation

**Figure 1.**
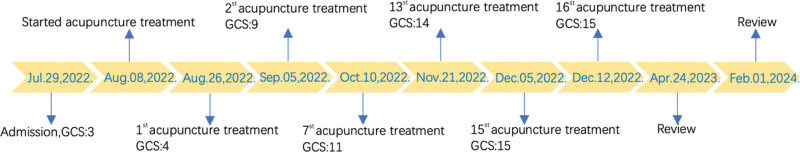
Timeline.

The timeline with clinical and procedural data is shown in Figure 1.

**Figure 2.**
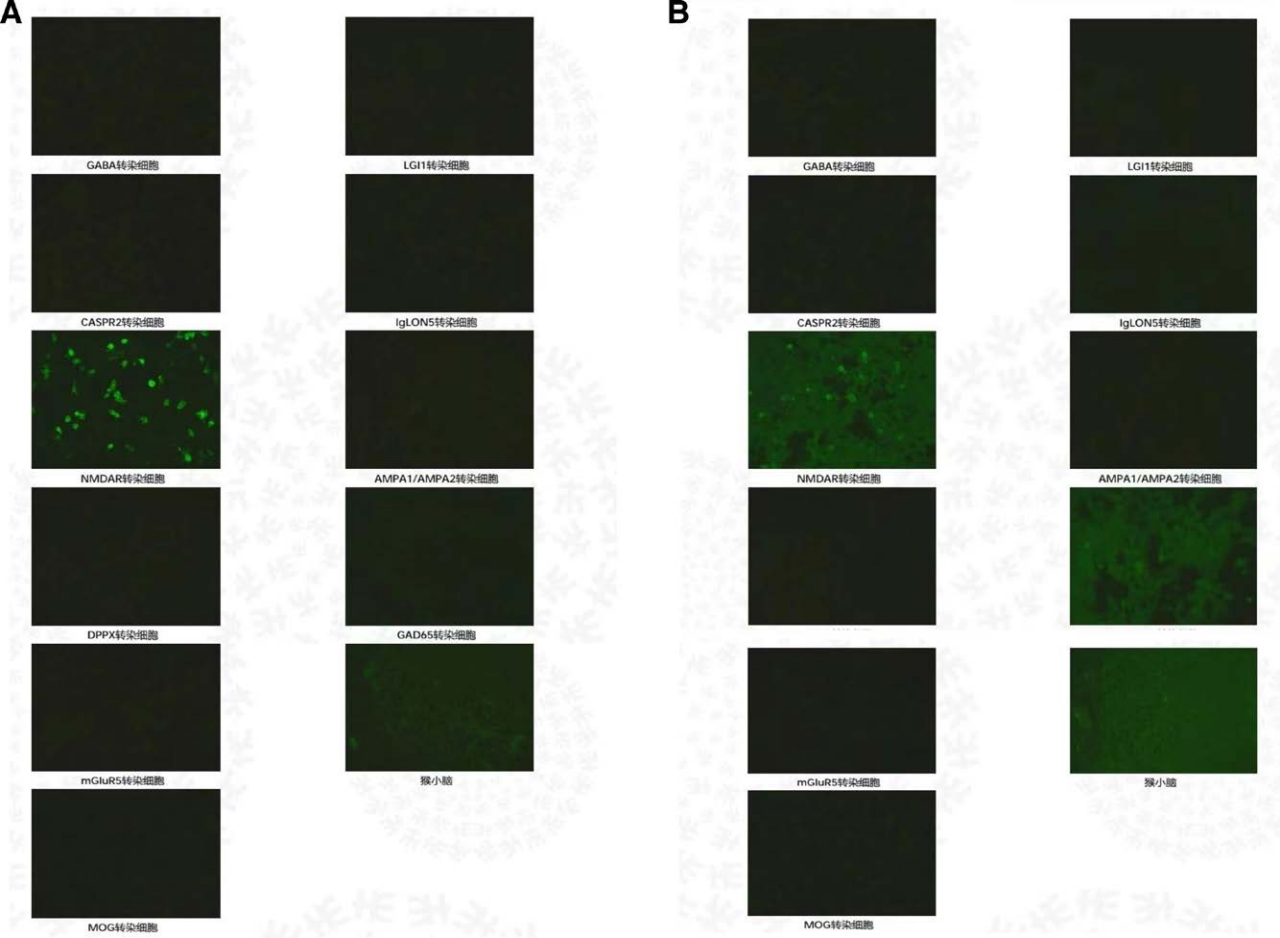
Results of NMDA antibody titers.

**Figure 3.**
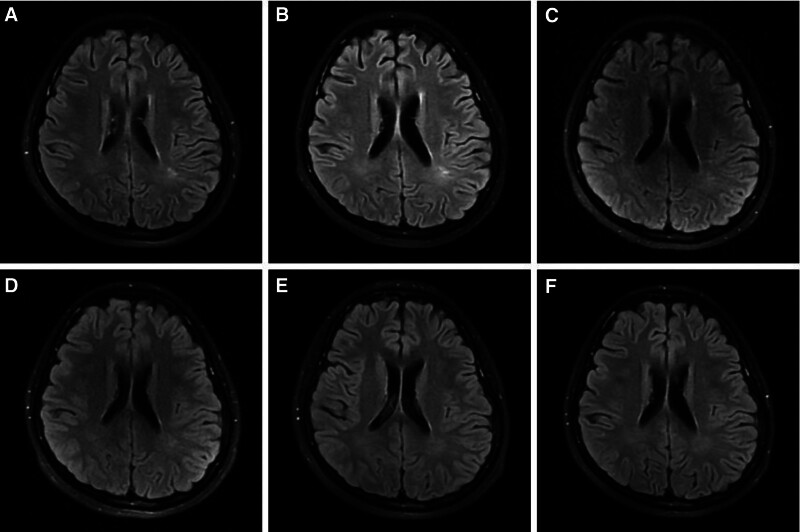
Results of cranial MRI.

We report a case of a 13-year-old girl who presented with fever, headache, and personality changes in July 2022. After receiving antiviral and symptomatic therapy, her condition worsened, and she became manic, experienced recurrent epileptic seizures, and went into a coma. She was referred to a higher-level hospital where the laboratory tests used indirect immunofluorescence assay suggesting that the titer of NMDA antibody in serum was 1:100 (Fig. 2A), and NMDA antibody in cerebrospinal fluid was 1:32 (Fig. 2B). The cerebrospinal fluid analysis suggested that the cerebrospinal fluid is slightly reddish and clear, with an erythrocyte count of 340*10^6^/L and elevated protein levels (415 mg/L↑). Other autoimmune encephalitis antibodies, p-ANCA, c-ANCA were within normal ranges. EEG showed persistent diffuse delta waves. Cranial MRI depicted abnormal signals in the white matter adjacent to the triangular region of the lateral ventricles bilaterally, diagnosed as “Anti-NMDAR encephalitis.” She was given 2 rounds of first-line immunotherapy (methylprednisolone pulse therapy + gamma globulin) and 1 round of second-line immunotherapy (intravenous rituximab). However, her symptoms did not relieve. She was admitted our hospital with a Glasgow Coma Scale (GCS) score of 3, fever, throat sputum, and intermittent seizures. She had a red tongue with a white greasy fur and a slippery pulse. Neurological examination suggested that physiological reflexes were present, and pathological reflexes were not elicited. Admission cranial MRI suggested a few flaky abnormal signal shadows in the left parietal lobe, with a slightly high signal on T2WI and FLAIR, and no significantly high signal on DWI (Fig. 3A). After 1 month of conventional treatment (including 3 rounds of rituximab), her temperature normalized and her seizures were controlled, but her state of consciousness did not improve. A repeat cranial MRI showed that the symptoms were roughly similar to the previous ones (Fig. 3B). The supervising physician decided to use acupuncture as an adjunctive therapy.

## 3. Acupuncture treatment

The patient diagnosis in Chinese medicine is “Shenhun.” The therapeutic principle was Kaiqiao Xingshen Huatan. The therapeutic method used was Xingnao Kaiqiao acupuncture. During the coma period, the major points were double Neiguan, Renzhong and double Sanyinjiao, and after the recovery of consciousness, the major points were double Neiguan, Yintang, Shangxing, and double Sanyinjiao. The matching acupoints were double Taichong, Zusanli, and Fenglong (Table 1). Each treatment lasted for 30 minutes. Five treatments were administered a week, and 4 weeks as 1 course.

**Table 1:** Acupuncture points.

Acupuncture points	Method of insertion
double Neiguan (PC6)	puncture straight for 10–15 mm, and apply the technique with lifting and twisting for 1 min.
Renzhong (DU26)	Puncture 3–5 mm obliquely toward the nasal septum, and apply with bird-peck diarrhea until the eye is moist.
Double Sanyinjiao (SP6)	Puncture obliquely along the medial border of the tibia at a 45° angle to the skin, and enter the needle 15–25 mm, to the extent of lifting and inserting and applying until the affected limb twitches 3 times.
Yintang (GV29)	Puncture diagonally at the root of the nose for 8–10 mm, using a light bird-pecking technique to the point of tearing or wetting the eyeballs.
Shangxing (DU23)	Puncture along the skin with a 75 mm long millipede needle, the tip of the needle penetrating to Baihui (DU20), applying small amplitude, high frequency, twisting for 1 min.
Double Zusanli (ST36)	Straight puncture 15–25 mm, administered by lifting and inserting for 1 min.
Double Taichong (LR3)	Straight puncture 10–15 mm, administered by lifting and inserting for 1 min.
Double Fenglong (ST40)	Straight puncture 15–25 mm, administered by lifting and inserting for 1 min.

## 4. Clinical results

**Figure 4.**
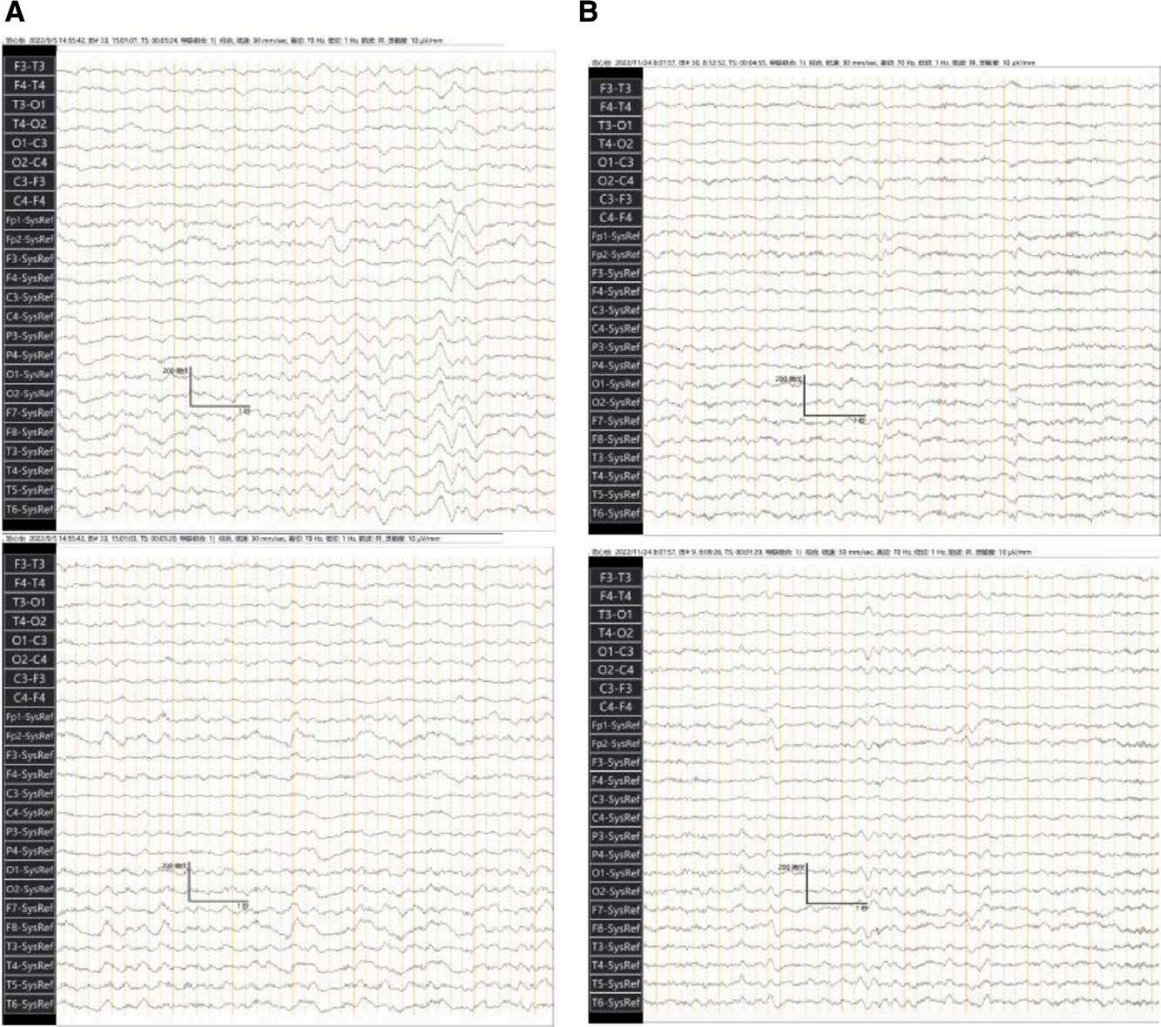
Results of electroencephalogram.

After 1 week of the above treatment, she could open her eyes when she was in pain, and visual tracking was observed. GCS score was 4. After 2 weeks, her eyes could be opened on call, tearing and retraction of limbs started with painful stimuli. GCS score was 9. A review of the EEG results suggested a high degree of abnormality (Fig. 4A). After 7 weeks, her consciousness was better than before, and she could make sounds, but speech was blurred and could not answer. GCS score was 11. After 13 weeks, she regained consciousness, and she was able to conduct simple conversations with clear articulation on her own. GCS score was 14. A review of cranial MRI showed a slightly high signal on the left parietal speckled FLAIR, and DWI did not show a significantly high signal (Fig. 3C). The new EEG suggested moderate height abnormalities (Fig. 4B). After 15 weeks, she was able to stand alone. After 16 weeks, the patient was able to walk independently, and communicate effectively with others. GCS score was 15.

After 16 weeks of acupuncture treatment, the patient clinical symptoms completely disappeared, and her level of consciousness, mobility, and speech were fully restored as normal. The discharge review of cranial MRI suggested that the left parietal lobe speckled FLAIR showed a slightly high signal, and DWI did not show an obvious high signal (Fig. 3D). Cranial MRI 4 months after discharge suggested that the original left parietal lobe with little abnormal signal was not observed (Fig. 3E). During a telephone follow-up 1 year after discharge, she recovered to normal, with no sequelae or recurrence. A review of cranial MRI showed no significant abnormal changes (Fig.3F).

The patient did not report any adverse reactions during the treatment, was satisfied with the effect of acupuncture, and confirmed its curative efficacy.

## 5. Discussion

NMDAR is an excitatory ionotropic glutamate receptor widely distributed in the frontotemporal lobe, hypothalamus, brainstem, and other parts of the brain. It is involved in the regulation of synaptic transmission, triggering synaptic remodeling and other processes, and is closely related to cognition, memory, and behavior.^[3]^ The organism generates an autoimmune response under viral infection, tumor, and other triggers, and the immune system produces memory B cells, which cross the blood-brain barrier to form antibodies.^[4]^ Antibodies bind to NMDAR and lead to internalization of the receptor and reduced function, resulting in a variety of clinical symptoms.^[5]^

The titer of NMDA antibody in the cerebrospinal fluid and the severity of clinical symptoms were found to be independent risk factors for poorer prognosis. Other studies have shown that a GCS score ≤ 8 is a risk factor for poor clinical outcomes in the short term (at discharge) and/or long term (3 months and 6 months after discharge).^[6,7]^ In this case, the patient symptoms did not improve with conventional treatment and had a high titer in the cerebrospinal fluid, with a GCS score of 4, suggesting a poor prognosis.

The “Xingnao kaiqiao” acupuncture technique was developed by academician Xuemin Shi in 1972, which is widely used in the treatment of various neurological disorders and reaps good therapeutic effects.

Many studies have shown that the “Xingnao kaiqiao” acupuncture technique can increase cerebral blood flow, promote the repair of nerve cells, and improve the cognitive and living abilities of patients. In addition, it can promote the repair and regeneration of cerebral nerve cells, increase the excitability of the brainstem reticular arousal system, release the inhibitory state of the cerebral cortex, and promote restoration of consciousness.^[8]^ Dr Zhongren Li et al found that acupuncture can increase the α wave index and amplitude of the EEG and reduce the abnormal β wave, q wave and d wave.^[9,10]^

The patient had regained consciousness after 16 weeks of acupuncture treatment, GCS score was 15. She was mobile, fluent in speech, and did not relapse at 1-year follow-up, which implies that acupuncture is an effective intervention to promote recovery in patients with anti-NMDAR encephalitis with impaired consciousness. It can be used as a complementary and alternative treatment for this disease with sustained improvement.

The following limitations exist in this study. This is a single case report and lacks a control group, the efficacy of acupuncture alone in the treatment of this disease cannot be adequately demonstrated when combined with conventional treatment. Therefore, a larger sample size with additional controls is needed to support the efficacy of acupuncture. However, acupuncture is a good adjunctive therapy for patients with poor results from conventional treatment.

## Acknowledgments

The authors would like to thank the patient and her guardians.

## Author contributions

**Conceptualization:** Tianhong Yang.

**Methodology:** Tianhong Yang.

**Writing – original draft:** Tianhong Yang.

**Writing – review & editing:** Tianhong Yang, Hui Cheng, Chunlei Gu.
